# Supplementary figures and images for: Classification of MLH1 Missense VUS Using Protein Structure-Based Deep Learning-Ramachandran Plot-Molecular Dynamics Simulations Method
Source: Int J Mol Sci. 2024 Jan 10;25(2):850. doi: 10.3390/ijms25020850 (PMC10815254; doi:10.3390/ijms25020850)

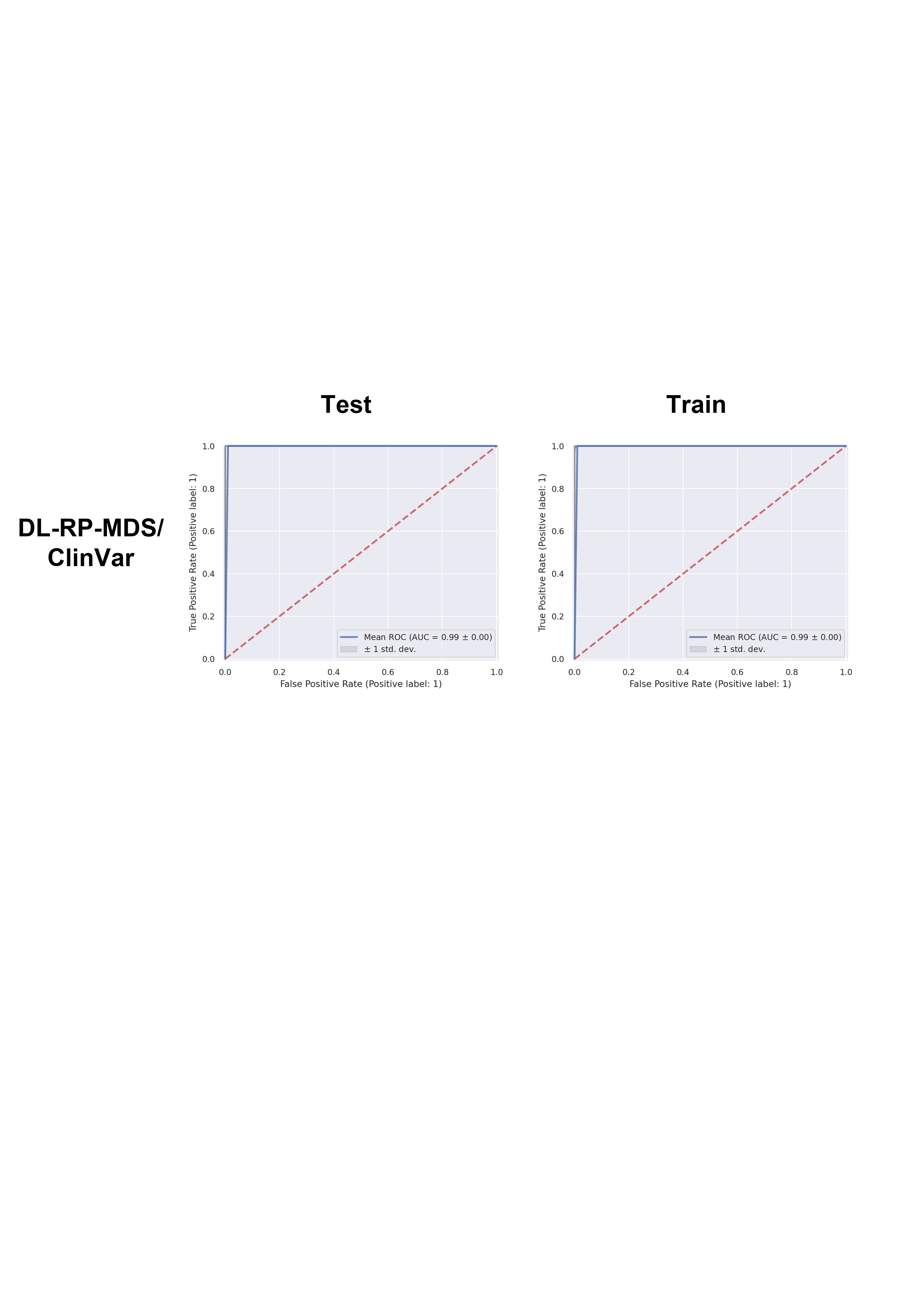

Supplement: Supplementary file 1 [file ijms-25-00850-s001.zip › Supplementary Figure S1.jpg]
